# P-757. Microbiological and clinical characteristics of extrapulmonary nontuberculous mycobacterial disease in a Chinese general hospital

**DOI:** 10.1093/ofid/ofae631.952

**Published:** 2025-01-29

**Authors:** Yumeng Yao, Rong Bao, Jue Pan, Bijie Hu

**Affiliations:** Zhongshan Hospital, Fudan University, Shanghai, Shanghai, China; Zhongshan Hospital, Fudan University, Shanghai, Shanghai, China; Zhongshan Hospital, Fudan University, Shanghai, Shanghai, China; Department of Infectious Diseases, Zhongshan Hospital, Fudan University, Shanghai, Shanghai, China

## Abstract

**Background:**

Data on extrapulmonary nontuberculous mycobacterial disease (ENTM) in China is lacking. We aim to explore the microbiological and clinical characteristics of ENTM in a general hospital.

**Methods:**

Laboratory extrapulmonary samples positive for nontuberculous mycobacteria in Zhongshan Hospital, Fudan University between Jan. 2017 and Dec. 2021 were retrospectively searched. The respective patient data were reviewed and followed until Dec. 2022. Thirty-three confirmed ENTM cases were included. Demographic, microbiological, clinical characteristics, as well as treatment and prognosis were analyzed.

**Results:**

A total of 33 confirmed ENTM cases were included. The average age was (49±16.3) years. Twenty-three cases (69.7%) were female and one third of the patients were immunocompromised. Skin and soft tissue infection, bone and joint infection, and disseminated NTM disease were the most common types (57.6%, 18.2%, and 15.6%). Healthcare-associated infections accounted for 51.5% (17/33) of the cases. *Mycobacterium abscessus* complex (42.4%) among rapidly growing mycobacteria (RGM) was the most common causative species. Patients received combination therapy with an average of (3.5±0.8) drugs for 6.3 (4.3,9.6) months. Most cases recovered (69.7%) or improved clinically (18.2%). Compared with RGM, infections with slowly growing mycobacteria (SGM) were seen in older individuals (60.2±15.0 vs 42.6±14.0, P<0.05) and were more often community-acquired (83.3% vs 28.6%, P<0.05).

**Conclusion:**

Skin and soft tissue infection is the most common type of ENTM, with *Mycobacterium abscessus* complex being the most important causative species. SGM infections affect older population and are more often community-acquired. Treatment of ENTM is challenging and lacks standardized protocol, future researches are warranted.

**Disclosures:**

**All Authors**: No reported disclosures

